# Effect of amniotic fluid stem cell transplantation on the recovery of bladder dysfunction in spinal cord-injured rats

**DOI:** 10.1038/s41598-020-67163-7

**Published:** 2020-06-22

**Authors:** Ching-Chung Liang, Sheng-Wen Steven Shaw, Yu-Shien Ko, Yung-Hsin Huang, Tsong-Hai Lee

**Affiliations:** 1 Female Urology Section, Department of Obstetrics and Gynecology, Chang Gung Memorial Hospital Linkou Medical Center, Taoyuan, Taiwan; 2 College of Medicine, Chang Gung University, Taoyuan, Taiwan; 3 Division of Obstetrics, Department of Obstetrics and Gynecology, Chang Gung Memorial Hospital Linkou Medical Center, Taoyuan, Taiwan; 4 Prenatal Cell and Gene Therapy Group, Institute for Women’s Health, University College London, London, UK; 5 The First Cardiovascular Division, Department of Internal Medicine, Chang Gung Memorial Hospital, Linkou Medical Center, Taoyuan, Taiwan; 6 Stroke Center and Department of Neurology, Chang Gung Memorial Hospital, Linkou Medical Center, Taoyuan, Taiwan

**Keywords:** Spinal cord diseases, Bladder

## Abstract

The effects of human amniotic fluid stem cell (hAFSC) transplantation on bladder function and molecular changes in spinal cord-injured (SCI) rats were investigated. Four groups were studied: sham and SCI plus phosphate-buffered saline (SCI + PBS), human embryonic kidney 293 (HEK293) cells, and hAFSCs transplantation. In SCI + PBS rat bladders, cystometry showed increased peak voiding pressure, voiding volume, bladder capacity, residual volume, and number of non-voiding contractions, and the total elastin/collagen amount was increased but collagen concentration was decreased at days 7 and 28. Immunoreactivity and mRNA levels of IGF-1, TGF-β1, and β3-adrenoceptor were increased at days 7 and/or 28. M2 immunoreactivity and M3 mRNA levels of muscarinic receptor were increased at day 7. M2 immunoreactivity was increased, but M2/M3 mRNA and M3 immunoreactivity levels were decreased at day 28. Brain derived-neurotrophic factor mRNA was increased, but immunoreactivity was decreased at day 7. HEK293 cell transplantation caused no difference compared to SCI + PBS group. hAFSCs co-localized with neural cell markers and expressed BDNF, TGF-β1, GFAP, and IL-6. The present results showed that SCI bladders released IGF-1 and TGF-β1 to stimulate elastin and collagen for bladder wall remodelling, and hAFSC transplantation improved these changes, which involved the mechanisms of BDNF, muscarinic receptors, and β3-adrenoceptor expression.

## Introduction

Spinal cord injury (SCI) affects approximately 2.5 million people worldwide and may cause severe physical disabilities^1^. Most patients with SCI develop neurogenic bladder symptoms, including overactive bladder, urinary retention, urinary tract infection, and chronic renal failure^2,3^. These neurogenic bladder conditions cause altered tissue morphology, such as bladder hypertrophy^4^ and fibrosis^5^, which adversely affect detrusor muscle function and result in bladder of low compliance and capacity^6^. Although the effects of SCI on bladder function have been extensively studied, changes in the mechanical behaviour of the SCI bladder wall tissue have not been fully established. Abnormal expression of collagen and elastin in human bladder muscle is a histological feature characteristic of noncompliant bladder^5,7^. In animal studies, bladder wall compliance is closely related to the collagen and elastin content^8,9^. In addition, insulin-like growth factor-1 (IGF-1) and transforming growth factor-beta 1 (TGF-β1) were reported to be involved in tissue remodelling and bladder wall fibrosis after SCI^9–11^.

Patients with chronic SCI and neurogenic bladder symptoms are currently treated with antimuscarinic medications, β3-adrenoceptor agonists, and bladder catheterization. However, these therapeutic methods are not effective and associated with adverse effects, such as dry mouth and constipation^12^. Recent research efforts have focused on attempts to regenerate the injured spinal cord using cell transplantation^13^.

A previous study showed that the treatment of SCI with umbilical cord blood stem cells could improve locomotor function and suggested that an increase in the levels of brain-derived neurotrophic factor (BDNF) and nerve growth factor (NGF) in the injured spinal cord is the main therapeutic mechanism of this effect^14^. Most studies of stem cell therapy in SCI have focused on motor limb functions and sensory recovery. However, impairment of quality of life from neurogenic bladder dysfunction exceeds that from the loss of limb function after SCI^15^.

Stem cell therapy for neurogenic bladder has been conducted mainly on an experimental basis in the area of bladder dysfunction^16^. Similar to embryonic stem cells, human amniotic fluid-derived stem cells (hAFSCs) are broadly multipotent. However, while embryonic stem cells are pluripotent, hAFSCs are not pluripotent but rather have the potential to differentiate into a variety of cell types^16,17^. Until now, except for the intraspinal transplantation of bone marrow mononuclear cells in acute transection-induced SCI in rats^18^, no studies have reported the therapeutic effect of hAFSCs on bladder function in SCI cystopathy. The purpose of the present study was to investigate the effects of hAFSC transplantation on bladder function and molecular changes in the bladder wall tissue of rats with SCI.

## Results

### Bladder weights of SCI rats improved after hAFSC transplantation

**Figure 1:**
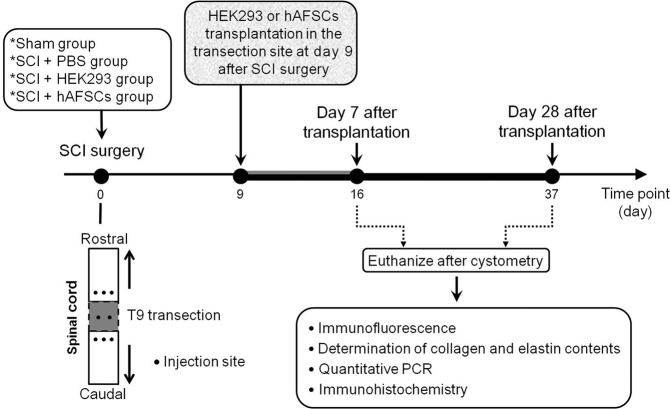
Schematic of the experimental procedure. SCI = spinal cord injury. PBS = phosphate-buffered saline. hAFSCs = human amniotic fluid stem cells. HEK293 = human embryonic kidney 293 cells. PCR = real-time polymerase chain reaction.

The body and bladder weights of sham and SCI rats that received transplantation of phosphate-buffered saline (SCI + PBS), human embryonic kidney 293 (HEK293) cells (SCI + HEK293), and hAFSCs (SCI + hAFSCs) were examined at day 9 after SCI surgery (Fig. 1) (Supplementary Table 1). After transplantation, the rat body weight was decreased at day 7 and showed a nonsignificant increase at day 28, but there was no significant difference in body weight among groups. However, rat bladder weight was significantly higher in the SCI + PBS and SCI + HEK293 groups than in the sham group at days 7 and/or 28, and hAFSC transplantation improved bladder weight at days 7 and 28 (P < 0.0001), although bladder weight did not return to the level in the sham group (Supplementary Table 1).

### Dysfunctions subsequent to SCI induction identified by cystometry improved after hAFSC transplantation

**Figure 2:**
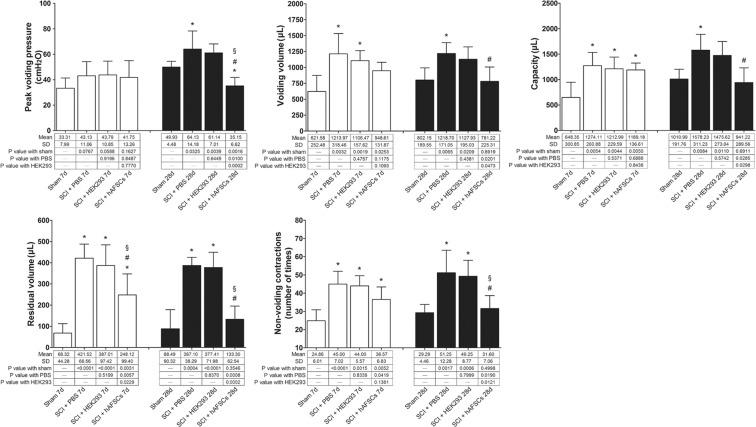
Cystometric studies after transplantation. The SCI + PBS group showed a significant increase in voiding volume, residual volume, bladder capacity, and non-voiding contraction at days 7 and 28. However, the peak voiding pressure of the bladders did not change at day 7 but was increased at day 28. The SCI + HEK293 group showed changes similar to those of the SCI + PBS group at days 7 and 28, except nonsignificant changes in peak voiding pressure, voiding volume and bladder capacity at day 28 after HEK293 cell transplantation. Dysfunction in these bladder parameters was recovered after hAFSC transplantation, but a significant increase in bladder capacity, residual volume and the number of NVCs at day 7 and a decrease in peak voiding pressure at day 28 remained. *P < 0.05 vs. sham group. ^#^P < 0.05 vs. SCI + PBS group. ^§^P < 0.05 vs. SCI + HEK293 group. N = 6 at each time point. HEK293 = human embryonic kidney 293 cells. PBS = phosphate-buffered saline.

Cystometry was performed in the sham group and the SCI + PBS, SCI + HEK293 and SCI + hAFSC groups at days 7 and 28 after SCI surgery (Fig. 2). The SCI + PBS group exhibited a significant increase in  voiding volume, residual volume, bladder capacity, and the number of non-vo iding contractions (NVCs) at days 7 and 28. However, the peak voiding pressure did not show a change at day 7 but was significantly increased at day 28. The SCI + HEK293 group showed changes similar to those of the SCI + PBS group at days 7 and 28, except changes in peak voiding pressure, voiding volume and bladder capacity at day 28 were nonsignificant. These dysfunctional bladder parameters were restored to near sham levels after hAFSC transplantation, but significant increases in bladder capacity, residual volume and the number of NVCs at day 7 and a decrease in peak voiding pressure at day 28 were still observed (Fig. 2).

### Bladder elastin and collagen level improved to the sham level after hAFSC transplantation

**Figure 3:**
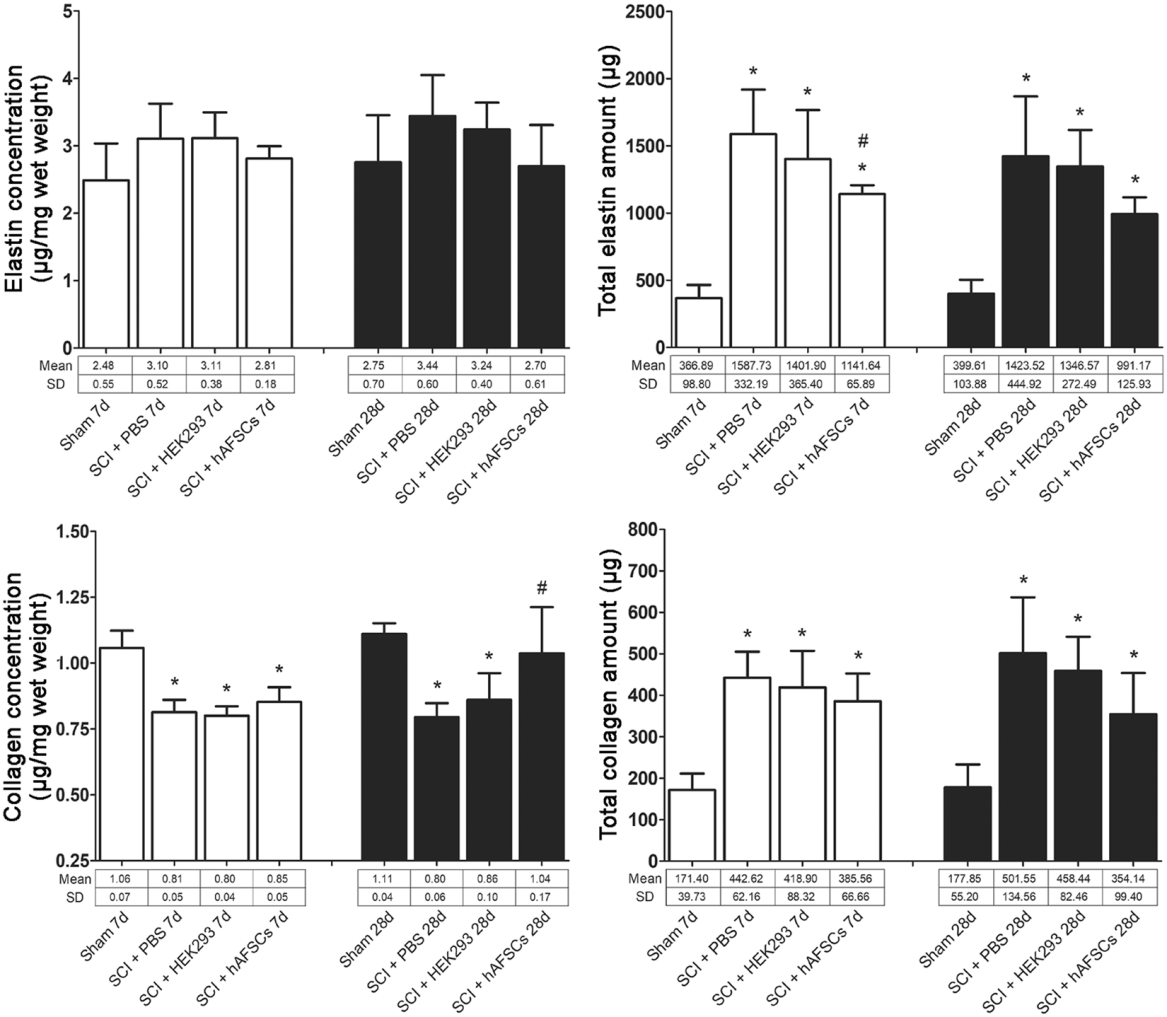
Concentration (μg/mg wet weight) and the total amount (μg) of elastin and collagen in the bladders of spinal cord-injured (SCI) rats after transplantation. The collagen concentration was decreased, and the total elastin and collagen amount was increased in the rat bladders of the SCI + PBS group at days 7 and 28. The SCI + HEK293 group showed the same changes as the SCI + PBS group at days 7 and 28. These changes improved to near sham levels, but a significant increase in total elastin and total collagen amount at days 7 and 28 and a decrease in collagen concentration at day 7 remained after hAFSC transplantation. *P < 0.05 vs. sham group. ^#^P < 0.05 vs. SCI + PBS group. N = 6 at each time point. HEK293 = human embryonic kidney 293 cells. PBS = phosphate-buffered saline.

At days 7 and 28 after transplantation, the total elastin and total collagen amount was significantly increased, but the collagen concentration was significantly decreased, whereas the elastin concentration showed a nonsignificant increase in the rat bladders in both the SCI + PBS and SCI + HEK293 groups (Fig. 3). These changes improved to near sham levels, but a significant increase in total elastin and total collagen amount at days 7 and 28 and a decrease in collagen concentration at day 7 were still observed after hAFSC transplantation (Fig. 3).

### Expression of TGF-β1 and IGF-1 improved after hAFSC transplantation

**Figure 4:**
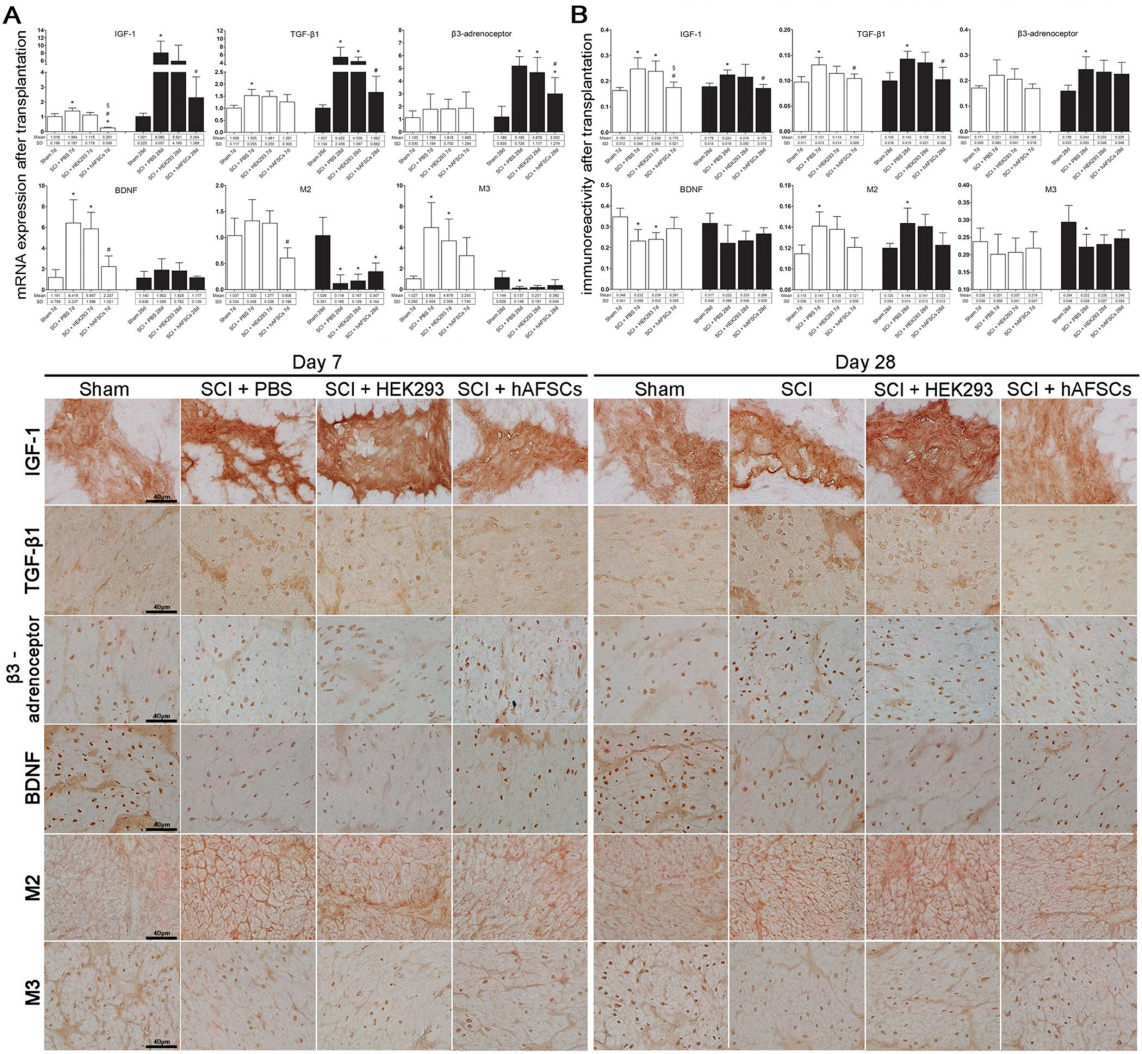
mRNA expression of IGF-1, TGF-β1, β3-adrenoceptor, BDNF, M2, and M3 in the bladders of spinal cord-injured (SCI) rats after transplantation. **(A)** The mRNA expression of IGF-1, TGF-β1, BDNF and M3 was increased in the SCI + PBS group at day 7. However, the mRNA expression of IGF-1, TGF-β1, and β3-adrenoceptor was increased, but that of M2 and M3 was decreased, in the SCI + PBS group at day 28. The SCI + HEK293 group showed a change similar to that of the SCI + PBS group but had no significant change in IGF-1 and TGF-β1 mRNAs at day 7 and IGF-1 and M3 at day 28. All the mRNA changes improved to near sham levels at days 7 and 28 except IGF-1, which was decreased at day 7; M2, which was decreased at day 28; and β3-adrenoceptor, which was increased at day 28 after hAFSC transplantation. Immunoreactivity of IGF-1, TGF-β1, β3-adrenoceptor, BDNF, M2, and M3 in the bladders of spinal cord-injured (SCI) rats after transplantation. **(B)** Immunoreactivity of IGF-1, TGF-β1, and M2 was increased, but BDNF was decreased in the SCI + PBS group at day 7. Immunoreactivity for IGF-1, TGF-β1, β3-adrenoceptor, and M2 was increased, but that for M3 was decreased in the SCI + PBS group at day 28. The SCI + HEK293 group showed a change similar to that of the SCI + PBS group, but no significant changes in TGF-β1 and M2 immunoreactivity at day 7 or in IGF-1, TGF-β1, β3-adrenoceptor, M2 and M3 immunoreactivity at day 28. These changes improved to near sham levels at days 7 and 28 after hAFSC transplantation. *P < 0.05 vs. sham group. ^#^P < 0.05 vs. SCI + PBS group. ^§^P < 0.05 vs. SCI + HEK293 group. N = 6 at each time point. BDNF = brain-derived neurotrophic factor. HEK293 = human embryonic kidney 293 cells. IGF-1 = insulin-like growth factor-1. M2 and M3 = M2 and M3 muscarinic receptors. PBS = phosphate-buffered saline. TGF-β1 = transforming growth factor-beta 1.

The immunoreactivity and mRNA levels of IGF-1 and TGF-β1 in the bladder were significantly increased in the SCI + PBS group at days 7 and 28 after transplantation, but only IGF-1 immunoreactivity at day 7 and TGF-β1 mRNA levels at day 28 were significantly increased in the SCI + HEK293 group (Fig. 4). However, all these changes improved after hAFSC transplantation (Fig. 4).

### Expression of β3-adrenoceptor, muscarinic receptors, and BDNF improved after hAFSC transplantation

The immunoreactivity and mRNA levels of β3-adrenoceptor were increased in the rat bladders in the SCI + PBS group at day 28 (Fig. 4). The M2 immunoreactivity and M3 mRNA levels of muscarinic receptors were increased in the rat bladders in the SCI + PBS and/or SCI + HEK293 groups at day 7. However, unlike M2 immunoreactivity, which was increased, M2/M3 mRNA and M3 immunoreactivity levels were primarily decreased in the SCI + PBS group at day 28. BDNF showed an increase in mRNA expression but a decrease in immunoreactivity at day 7, and no significant change in BDNF mRNA expression or immunoreactivity at day 28 was observed in either the SCI + PBS or SCI + HEK293 group. Except for the decrease in IGF-1, all these changes improved after hAFSC transplantation (Fig. 4).

### Expression of the neural cell markers including human CD90 (hCD90), nestin, β-Tubulin III and GFAP in cultured hAFSCs

**Figure 5:**
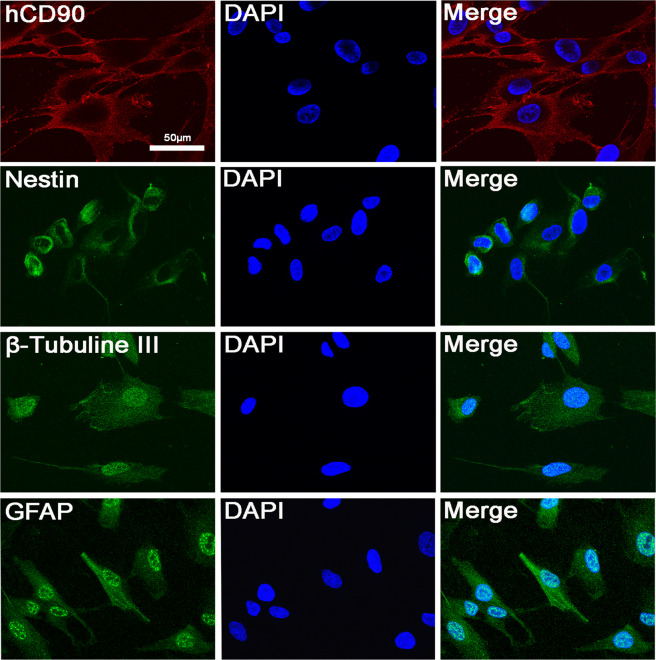
Demonstration of the neural nature of hAFSCs with immunoconfocal microscopy in hAFSC culture. Colocalization (merge) between the DAPI and neural cell markers (hCD90, nestin, β-tubulin III and GFAP) signals is shown. Bar in hCD90 = 50 μm. hCD90 = human CD90. GFAP = glial fibrillary acidic protein. DAPI = 4′,6-diamidino-2-phenylindole.

The neural nature of cultured hAFSCs was examined with immunoconfocal microscopy (Fig. 5). The results showed colocalization of the DAPI signal with signals for the neural cell markers including hCD90, nestin, β-Tubulin III and glial fibrillary acidic protein (GFAP) (Fig. 5).

### Anti-autoimmunity and anti-inflammatory effects of hAFSCs

**Figure 6:**
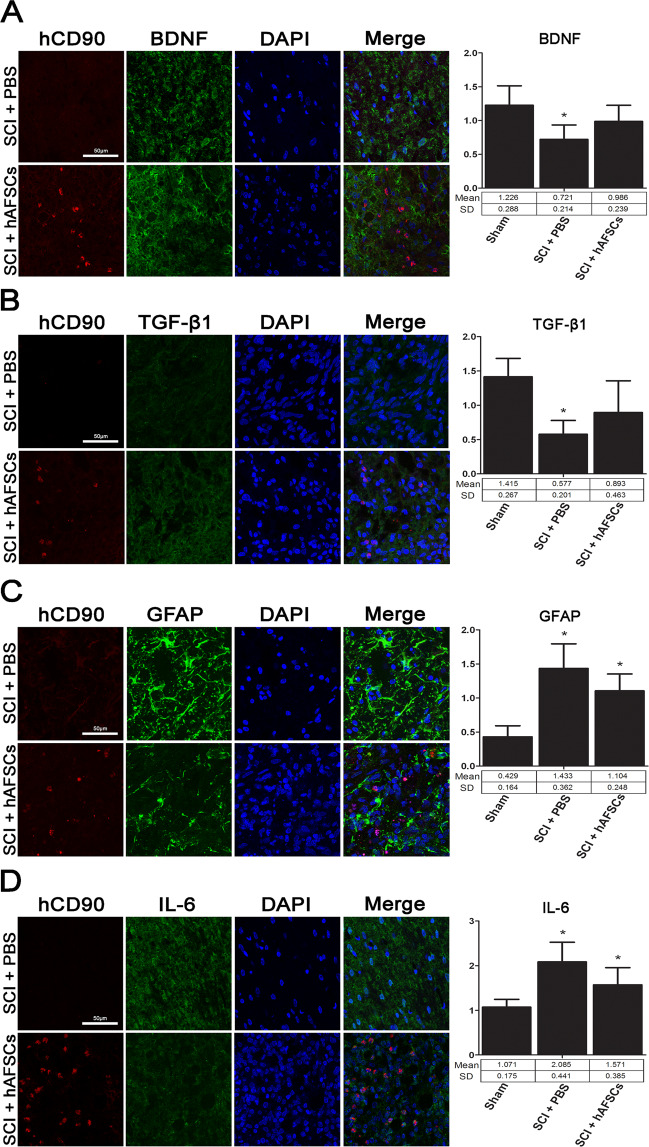
Immunoconfocal microscopy using the triple labelling technique. Colocalization (merge) of DAPI and hCD90 with the neural cell markers BDNF, TGF-β1, GFAP, and IL-6 was observed in spinal cord-injured (SCI) sections after transplantation. Immunofluorescence expression of BDNF (**A**) and TGF-β1 (**B**) was reduced, but GFAP (**C**) and IL-6 (**D**) was significantly increased in the SCI + PBS group compared to the sham group. These changes improved after hAFSC transplantation. Bar in the hCD90 panel = 50 μm. N = 6 at each time point. hCD90 = human CD90. BDNF = brain-derived neurotrophic factor. TGF-β1 = transforming growth factor-beta 1. GFAP = glial fibrillary acidic protein. IL-6 = interleukin-6. DAPI = 4′,6-diamidino-2-phenylindole.

**Figure 7:**
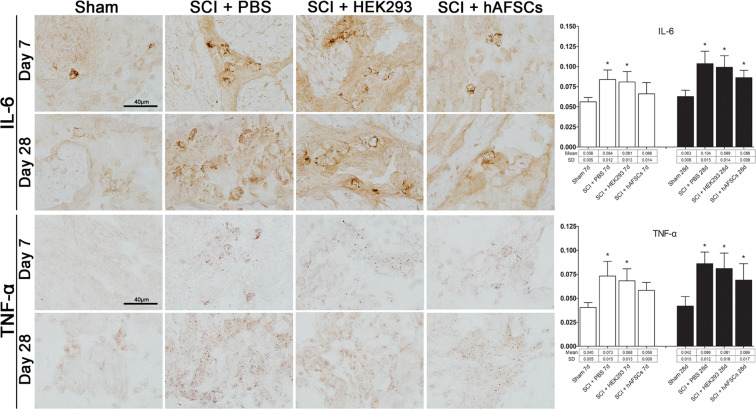
Immunostaining for IL-6 and TNF-alpha in the bladder sections of sham rats (Sham) and spinal cord-injured (SCI) rats after phosphate-buffered saline (SCI + PBS), HEK293 cells (SCI + HEK293) and hAFSC (SCI + hAFSC) transplantation. Compared to that in the sham group, the expression of IL-6 and TNF-alpha in bladder tissue was significantly increased in the SCI + PBS and SCI + HEK293 groups. hAFSC transplantation recovered IL-6 and TNF-alpha to near sham levels at day 7, but less recovery was observed at day 28. Bar in the sham panel = 40 μm. *P < 0.05 vs. sham group. N = 6 at each time point. HEK293 = human embryonic kidney 293 cells. IL-6 = interleukin-6. TNF-alpha = tumor necrosis factor-alpha.

The effects of hAFSCs in spinal cord sections from SCI rats at day 7 were examined using immunoconfocal microscopy with triple labelling (Fig. 6 and Supplementary Figure S1). The results demonstrated the colocalization of DAPI and hCD90 with the expression of BDNF, TGF-β1, GFAP, and interleukin-6 (IL-6) in hAFSCs, indicating that hAFSCs could produce neurotrophic factors and possess anti-autoimmunity and anti-inflammatory effects. The expression of BDNF and TGF-β1 was significantly decreased, but the expression of GFAP and IL-6 was significantly increased in the SCI + PBS group compared to the sham group, and all of these parameters improved at 7 days after hAFSC transplantation (Figs. 6 and 7 and Supplementary Figure S1).

The effects of hAFSCs were also examined in the bladder sections of SCI rats at days 7 and 28 using immunohistochemistry (Fig. 7). Compared to that in the sham group, the expression of IL-6 and tumor necrosis factor-alpha (TNF-alpha) in bladder tissue was significantly increased in the SCI + PBS and SCI + HEK293 groups. hAFSC transplantation restored IL-6 and TNF-alpha expression to near sham levels at day 7, but less pronounced recovery was observed at day 28.

### Fate of hAFSCs transplanted into the injured spinal cord

The engraftment effect of hAFSCs in spinal cord sections from SCI rats was evaluated by immunoconfocal microscopic analysis with antibody against hCD90 (Supplementary Figure S2). While the sham control was negative for hCD90 staining, hCD90-positive hAFSCs were present at days 3, 7 and 14 after hAFSC transplantation, and the number of hCD90-positive hAFSCs was significantly lower at day 14 than at days 3 and 7 (Supplementary Figure S2).

## Discussion

**Figure 8:**
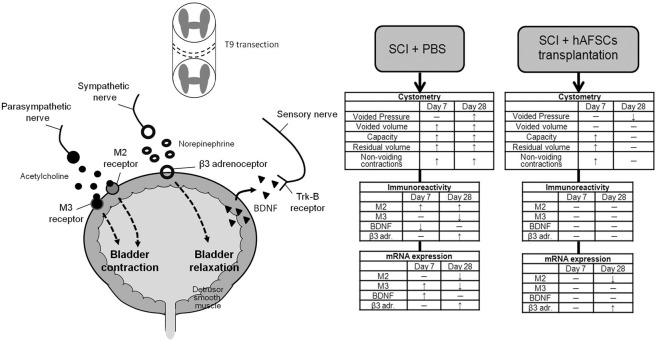
Schematic diagram of the peripheral neurotransmitters and BDNF, which are responsible for bladder contraction and relaxation. The mechanism of bladder dysfunction induced by spinal cord injury (SCI) in rats was found to involve M2, M3, β3-adrenoceptor receptor, and BDNF in the bladder. Bladder dysfunction improved after hAFSC transplantation.

A summary of the results and possible mechanisms of hAFSC transplantation in SCI rats is shown in Fig. 8. The present study showed that in SCI rats, initial/final body weight improved at day 28, and bladder weight did not return to the level in the sham group at days 7 and 28, but bladder function improved after hAFSC transplantation. The increase in bladder weight may be due to increased bladder wall muscle and urothelium^19^. Additionally, connective tissue and fibrotic changes accumulated in the bladder walls, which adversely affected detrusor function and the bladder's capacity to empty^5^ and might result in irreversible bladder damage even after hAFSC transplantation.

Alterations in collagen and elastin cause morphologic changes in the bladder that affect its contractility. In this study, the total bladder collagen and elastin amount was significantly increased at days 7 and 28 after SCI. Kadekawa *et al*.^20^ observed increases in collagen mass at 4–12 weeks and elastin mass at 2–12 weeks in SCI rats. Similar to our results, Wada *et al*.^9^ found that total collagen and elastin at week 4 were significantly increased in the bladders of rats after SCI compared to the bladders of healthy rats with no SCI. In a previous study, the relative collagen concentration was significantly decreased, and the relative elastin concentration was significantly increased in bladders at week 3 after SCI compared to healthy rat bladders^21^. This increase in elastin is suggested to cause decreased stress relaxation of SCI bladders within the first 2–3 weeks of SCI^21,22^, whereas the collagen concentration after SCI is decreased due to increased bladder mass secondary to detrusor hypertrophy^8^. Our data showed that the collagen concentration was decreased, but the elastin concentration was increased in SCI bladders; however, both improved to sham levels at day 28 after hAFSC transplantation. Another study showed that bladder wall compliance in SCI rats was significantly greater at weeks 3–6 after SCI compared to that of normal bladders, but at week 10, this compliance had been decreased to a level near that of the normal bladders^8^. These results indicate that SCI and associated changes in the mechanical environment of the SCI bladder may trigger the synthesis of IGF-1 and TGF-β1 in detrusor tissue, which stimulates elastogenesis^22^.

The fibrosis markers IGF-1 and TGF-β1 could induce hypertrophy and upregulate collagen in bladder smooth muscle cell cultures^23^. The mRNA and protein levels of IGF-1 and TGF-β1 were significantly increased in the bladders of SCI rats, which suggests that fibrosis can change bladder wall compliance^21,22^. The mRNA levels of TGF-β1 were significantly increased at weeks 1, 2, 8, and 12 after SCI, and types 1 and 3 collagen levels were significantly higher in rat bladders at weeks 1, 2, and 4 after SCI compared to those in the normal group^20^. Nagatomi *et al*.^22^ reported that mRNA levels of both IGF-1 and TGF-β1 increased within 3 days after SCI, but the mRNA levels of only TGF-β1 remained significantly increased at day 7 and week 3 after SCI. Although IGF-1 and TGF-β1 protein levels in the bladders of SCI rats were quantified, the level of only TGF-β1 was significantly higher than that in healthy rats at day 3 and week 3 after SCI. However, IGF-1 protein levels were upregulated at day 3 but had returned to normal at week 3 after SCI. We found that IGF-1 and TGF-β1 immunoreactivity and mRNA levels were significantly increased in the bladders of SCI rats, but the expression levels of both were reduced, and bladder dysfunction had improved by days 7 and 28 after hAFSC transplantation.

SCI above the lumbosacral level interrupts the coordinated activity of the bladder and external urethral sphincter, which leads to NVC and detrusor-sphincter dyssynergia, impedes micturition, and results in a large volume of residual urine^24^. Moreover, bladder fibrosis induced by collagen deposition can also reduce bladder compliance and voiding efficiency^5^. Reduced voiding efficiency causes elevated urine volumes and increased bladder capacity in SCI rats^25^. Yoshiyama *et al*.^25^ reported that although SCI rats had a larger voiding pressure and voiding volume, the residual volume at week 3 was higher in rats after SCI compared to normal control rats. Cystometric analyses of SCI rats showed significant increases in the number of NVCs and in bladder capacity at weeks 2, 8, and 12 after SCI compared to those in the normal group^20^. NVCs in SCI rats are mediated by C-fibre afferent nerves^26^. In this study, peak voiding pressure was increased at day 28 but unchanged at day 7 after SCI, which reflects serial changes in bladder compliance. Analysis of other cystometric parameters in SCI rats showed increases in voiding volume, bladder capacity, residual volume, and NVCs at days 7 and 28 after SCI, and all of these parameters had improved by day 28 after hAFSC transplantation.

Significant improvements in bladder dysfunction after cell transplantation are attributed to attenuated external urinary sphincter function and reduced bladder outlet pressure due to improved neuronal circuitry^27^. A meta-analysis evaluating bladder recovery after stem cell-based therapy in SCI patients showed significant improvements in voiding pressure, residual volume, and NVC^6^. Preclinical SCI studies have used various types of stem cells for transplantation to treat bladder dysfunction caused by SCI, such as neural stem cells^28^, neuronal-glial restricted precursor cells^27,29^, and bone marrow stromal cells^27,30^. Voiding pressure and post-void residual volume significantly improved after neural stem cells were transplanted into the injured rat spinal cord^31^. Temeltas *et al*.^27^ demonstrated that bladder capacity, voiding pressure, voiding volume, and post-void residual volume significantly improved in SCI rats transplanted with neuronal-glial restricted precursor cells or bone marrow stromal cells compared with SCI controls. Hu *et al*.^30^ found that intravenous transplantation of bone marrow stromal cells could promote the recovery of lower urinary tract function in SCI rats. Voiding pressure, episodes of NVC, and residual volumes were significantly decreased in SCI rats treated with bone marrow stromal cells compared to controls^30^.

Bladder muscle is rich in muscarinic receptors, most of which are of the M2 subtype, while the rest are of the M3 subtype^32^. Muscarinic receptors of both the M2 and M3 subtypes are involved in mediating the contraction of bladder smooth muscle^33^. The mechanism underlying regulation of the bladder following SCI by muscarinic receptors is not fully understood. Normal detrusor contractions are thought to be mediated by the M3 subtype, whereas in patients with SCI and congenital myelomeningocele, detrusor contractions are mediated by the M2 subtype^34^. In an animal study, immunoreactivity of the M2 receptor in bladders of non-voiding SCI rats was significantly higher than that of the control group, but M3 immunoreactivity was not different compared to that in the control group^35^. Our results showed that the mRNA levels of M2 and M3 muscarinic receptors in the bladders of SCI rats were higher at day 7 (M2, nonsignificant difference) but significantly lower at day 28. These rat bladders had increased M2 immunoreactivity at days 7 and 28 but decreased M3 immunoreactivity at days 7 (nonsignificant) and 28. During the voiding phase, acetylcholine activates M2 receptors to facilitate bladder contraction by reducing adenylyl cyclase activity^33^. In addition, M2 receptors were reported to be involved in the inhibition of β3-adrenoceptor-induced detrusor relaxation^36^. During the storage phase of micturition, norepinephrine released from sympathetic nerves interacts with β3-adrenoceptors on the bladder wall to produce detrusor relaxation^37^. In this study, immunoreactivity and mRNA levels of β3-adrenoceptor in the bladders of SCI rats were significantly increased at day 28 but improved after hAFSC transplantation.

BDNF is a mediator of bladder dysfunction after SCI via the inhibition of neuronal sprouting^38^. A few studies have shown changes in endogenous BDNF in the bladders of SCI rats, but their results were inconsistent^39–41^. Vizzard^40^ found that BDNF mRNA levels in SCI bladders were increased at day 4 or week 4 after spinal cord transection, but the BDNF protein did not exhibit this change. In a contused spinal cord rat model, BDNF protein levels in the bladder at day 4 after SCI were slightly higher than those in normal rats. BDNF levels were then decreased over time to less than the normal range at day 21 after SCI^41^.

Our results showed expression disparity, as M3 and BDNF immunoreactivities were decreased, whereas their mRNA levels were increased mainly at day 7, and M2 immunoreactivity was increased, whereas its mRNA level was decreased mainly at day 28 in the rat bladders in the SCI + PBS and/or SCI + HEK293 groups. This expression disparity was also seen for NGF protein and mRNA levels after SCI-induced cystitis and was suggested to reflect retrograde axonal transport of NGF to the dorsal root ganglia, which may be related to altered lower urinary tract function^40^. Our previous study^42^ also demonstrated expression disparity between BDNF immunoreactivity and mRNA in ischaemic hippocampal neurons, which might be related to delayed neuronal death. The expression disparity of M2, M3 and BDNF between protein and mRNA levels likely indicates the occurrence of cellular dysfunction due to mRNA transcript instability, decreased translation efficiency, aberrant posttranslational mechanisms and retrograde axonal transport^40^, but hAFSC transplantation improved this expression disparity.

We found that hAFSC transplantation effectively reduced BDNF mRNA levels to near sham levels at day 7, but there was no difference in BDNF mRNA levels between the SCI + hAFSC group and the SCI + PBS group at day 28. Park *et al*.^39^ reported that human mesenchymal stem cell transplantation did not effectively improve bladder dysfunction in spinal cord-contused rats, and BDNF levels in these rat bladders were not increased at days 28 and 56 after stem cell transplantation. Our study found that HEK293 cell transplantation did not improve bladder function in SCI rats, as was observed for PBS. A previous study showed that the treatment of SCI with transplanted human umbilical cord blood stem cells improved locomotor function, suggesting that the increased levels of BDNF, NGF, and neurotrophin-3 in the injured spinal cord were responsible for the main therapeutic effects^14^.

In the present study, we examined the expression of BDNF, TGF-β1, GFAP, and IL-6 in spinal cord sections from SCI rats (Fig. 6 and Supplementary Figure S1) and IL-6 and TNF-alpha in bladder sections from SCI rats (Fig. 7). Our study demonstrated that hAFSCs had the capability to produce BDNF and possessed anti-autoimmunity and anti-inflammatory functions. However, we found that although the number of hAFSC decreased to nearly zero at day 14 (Supplementary Figure S2), hAFSC transplantation could still improve bladder dysfunction at day 28 (Fig. 2). This phenomenon may suggest that the mechanisms of functional improvement following SCI may not be mainly related to neurogenesis caused by hAFSC differentiation but are related to multimodal actions. A previous report found that hAFSCs could be integrated into various brain areas and migrate away from the graft area to become mature neurons and oligodendrocytes^43^. The concept of the functional multipotency of mesenchymal stem cells^44^ suggests that in addition to their ability to shed secretomes and exosomes that may contain growth factors, anti-inflammatory proteins, membrane receptors and microRNAs, stem cells may undergo chemotactic migration towards developmental targets or inflammatory areas^44^. The decreased number of hAFSCs at day 14 may suggest that hAFSCs have largely differentiated into mature neurons and oligodendrocytes or chemotactically migrated to the injured areas. Since our immunoconfocal study was performed in sections with relatively preserved tissue located around the injured spinal cord, it is possible that a larger number of hAFSCs were present in the injured areas of the spinal cord at day 14.

The present study has some limitations. First, we used hAFSCs at a single density. It is possible that a higher density or repeated transplantation could have better effects on SCI-induced bladder dysfunction. Second, we examined functional and morphological alterations in the bladders of SCI rats at days 7 and 28 after transplantation, but better results could be obtained if cystometric analysis was performed at a longer period after transplantation. Third, we did not directly examine cellular differentiation after hAFSC transplantation into bladders. However, we identified the expression of neural cell markers including hCD90, nestin, β-Tubulin III and GFAP in hAFSC cultures. A previous report revealed that hAFSCs could be induced towards neural differentiation, and the specific markers GFAP, beta-III tubulin, CNPase, MAP2, NeuN, synapsines, S100, and PMP22 could be detected^43^. It is likely that hAFSCs can differentiate and integrate into nervous tissue and may have therapeutic potential in treating neurological disorders. Fourth, due to the complex nature of SCI cystopathy, further study to elucidate the major site of action and long-term efficacy of hAFSC transplantation may be necessary. Fifth, we did not perform any type of immunosuppressive study. Our study and previous studies have revealed that injection of hAFSCs in animal models developed no immune rejection against transplanted tissue^17,45,46^. Mesenchymal stem cells have the advantage of immunomodulatory properties, making these cells good candidates for xenotransplantation^47^. Our study has demonstrated that hAFSCs share some characteristics with mesenchymal stem cells, such as immune modulations, so hAFSCs may not exhibit immune rejection, unlike haematopoietic stem cells. Sixth, we used HEK293 cells as the transplantation control for hAFSCs instead of fibroblasts. Fibroblast has been used as the transplantation control compared to human mesenchymal stem cell in SCI rats, and the results showed BDNF and neurotrophin-3 levels in spinal cord and bladder were not different between stem cell and control groups regardless the experimental duration^39^. HEK293 was reported to possess receptors for fibroblast growth factor^48^ but has limited neuroprotective effects. To prevent our study purpose from being misleading, we used HEK293 cells as a transplantation control.

In conclusion, functional changes in the SCI bladder may be related to the release of IGF-1 and TGF-β1, which stimulates collagen and elastin remodelling, leading to alterations of biomechanical properties in bladder wall tissue. Our data support the finding that SCI-induced bladder dysfunction can be improved by hAFSC transplantation, which may be related to an improvement in β3-adrenoceptor, BDNF, and muscarinic receptors.

## Methods

### Animals

Animal experiments were approved by the Institutional Animal Care and Use Committee of Linkou Chang Gung Memorial Hospital (No. 106–0299 C) and followed the National Institute of Health Guide for the Care and Use of Laboratory Animals (NIH publications No. 80–23) revised in 1996. Female Sprague-Dawley rats (10–12 weeks old) were given tap water ad libitum and maintained in a temperature- and humidity-controlled room on a 12-h light/dark cycle.

### Experimental design

Four groups were assigned: (1) sham rats, which underwent laminectomy without SCI (sham); (2) SCI rats treated with PBS (SCI + PBS); (3) SCI rats transplanted with HEK293 cells (SCI + HEK293); and (4) SCIrats transplanted with hAFSCs (SCI + hAFSCs). Conscious cystometry was performed at days 7 and 28 after transplantation (n = 6 at each time point). Animals were euthanized after cystometry, and bladders were transected at ureteral orifices and weighed. Total collagen and elastin amount and concentration in each bladder was determined using biochemical assays. The expression of IGF-1, TGF-β1, β3-adrenoceptor, BDNF, and the M2 and M3 muscarinic receptors in the bladder was measured by immunohistochemistry and real-time polymerase chain reaction (PCR). To assess hAFSC biological features and fate, immunoconfocal microscopy was performed against an undifferentiated neural cell marker (nestin), an immature neuronal marker (β-tubulin III), and an astroglial marker (GFAP) in hAFSC cultures and against BDNF, TGF-β1, GFAP, and IL-6 in SCI sections, and immunostaining against IL-6 and TNF-alpha in bladder tissue was performed. The experimental procedure is presented in Fig. 1.

### hAFSC isolation and characterization

hAFSCs were obtained from the fresh amniotic fluid of a single healthy pregnant donor after informed consent had been obtained. Cells were cultured in StemPro MSC serum-free medium supplemented with 10% foetal bovine serum (Invitrogen, Carlsbad, CA, USA) and incubated at 37 °C in room air with 5% CO_2_. Surface antigens specific of hAFSCs were characterized using a FACSCalibur flow cytometer (Becton Dickinson, Heidelberg, Germany)^17^. Similar to our previous publication^49^, the cells were positively stained with phycoerythrin-conjugated antibodies against CD44, CD45, CD73, CD90, CD105, and CD117 (BD PharMingen, San Diego, CA, USA) and positive for C-kit. They were capable of differentiating into several lineages based on the expression of markers after appropriate differentiation protocols. After 6–8 passages, hAFSCs were prepared at a density of 1 × 10^6^ cells/50 µL in PBS. The transplantation dose was determined following our previous study, in which hAFSCs were used to treat bladder dysfunction after focal cerebral ischaemia in rats^17^.

### HEK293 cell culture

Human embryonic kidney 293 cells (HEK293, American Type Culture Collection, Rockville, MD, USA), a transfected cell line of kidney origin, were used as a transplantation control. HEK293 cells were cultured in Glasgow MEM supplemented with 10% foetal bovine serum (Gibco, Invitrogen, Carlsbad, CA, USA) and 1% penicillin-streptomycin and incubated at 37 °C with 5% CO_2_. Cells at passage 10–11 were prepared at a density of 1×10^6^ cells/50 μL in PBS. The transplantation dose was the same as that of hAFSCs.

### SCI surgery

Under isoflurane anaesthesia, laminectomy was performed at T9, and the spinal cord was transectioned with a microscissors. Retraction of the upper and lower segments of the transected cord was carried out to confirm complete transection of the spinal cord. Animals were housed in separate cages until they had recovered from anaesthesia and were nursed twice daily with gentle massages of the bladder until the micturition reflex was restored.

### Donor cell transplantation

HEK293 cells or hAFSCs were transplanted at day 9 after SCI^31,39^. PBS, HEK293 cells or hAFSCs were injected in the following three locations using a Hamilton microsyringe (Hamilton Company, Reno, NV, USA) with 27 G needle: (1) 1 mm rostral to the SCI site (3 points), (2) 1 mm caudal to the SCI site (3 points), and (3) the SCI site (4 points) (Fig. 1). At each point, 5 μL of PBS, HEK293 cells or hAFSCs was infused for 60 sec, for a total of 50 µL PBS, 50 µL HEK293 cells or 1 × 10^6^ hAFSCs at 10 points. Following injection, the syringe was held in place for an additional 10 sec before removal to prevent the outflow of injected cells.

### Cystometric study

Rats underwent suprapubic tube implantation under 3% isoflurane inhalation 2 days before cystometry. Conscious cystometric studies were performed with the rats placed in metabolic cages (Med Associates, Saint Albans, VT, USA). Five cystometric parameters, peak voiding pressure, voiding volume, bladder capacity, residual volume, and non-voiding contraction, were collected over 5 micturition cycles for analysis with Cystometry Analysis version 1.05 (Catamount Research and Development, Saint Albans, VT, USA).

### Collagen and elastin quantification

The collagen and elastin quantities in each bladder were measured by dye-binding methods (Sircol collagen and Fastin elastin assay kits, Biocolor, Ltd., Carrickfergus, UK)^9^. After the total amounts of collagen and elastin per bladder were evaluated, concentrations were calculated as the total amount divided by bladder tissue weight (µg/mg wet weight).

### Quantitative real-time RT-PCR of the bladder

According to the manufacturer’s protocol, total RNA was prepared using TRIzol reagent (Invitrogen, Carlsbad, CA, USA) and incubated in a reverse transcription mixture at 25 °C for 5 min, 50 °C for 1 h, and 70 °C for 15 min before cooling to 4 °C for 5 min. Transcripts encoding for IGF-1, TGF-β1, β3-adrenoceptor, BDNF, M2, and M3 were measured using a TaqMan Universal PCR Master Mix Kit (Applied Biosystems, Foster City, CA, USA) and an ABI Prism 7900 sequence detection system (Applied Biosystems, Oster City, CA, USA). The IGF-1, TGF-β1, β3-adrenoceptor, BDNF, M2 and M3 assay codes were Rn00572010-m1, Rn00710306-m1, Rn00565393-m1, Rn02531967-s1, Rn02532311-s1, and Rn00560986-s1, respectively (Applied Biosystems, Oster City, CA, USA). The GAPDH expression (code Rs99999916-s1) was used as an endogenous control to allow for the quantification of relative gene expression. The PCR conditions were 50 °C for 2 min, 95 °C for 10 min, and 40 cycles at 95 °C for 15 sec and 60 °C for 1 min. The 18 S rRNA transcript was used as an internal control gene and amplified in a separate tube to normalize for variance in the input RNA. Fold change data were calculated with 2[Delta Delta C(T)]^17^. These values were summed and are expressed as the mean ± SD and compared between sham and each time point.

### Immunohistochemistry

Dissected bladders were fixed in optimal cutting temperature (OCT) compound, frozen in liquid nitrogen, and stored at −80 °C. Bladders were cryosectioned to yield 10 μm-thick slices at −20 °C. Fresh frozen sections were fixed for 10 min in acetone for the detection of IGF-1, TGF-β1, β3-adrenoceptor, BDNF, IL-6 and TNF-alpha and in 4% paraformaldehyde for detection of the M2 and M3 muscarinic receptors, air dried and then rinsed with PBS. Endogenous peroxidase was blocked by 0.2% H_2_O_2_ and 100% methanol for 10 min and washed with Dako REAL peroxidase blocking solution (code S2023, DAKO Corp., Carpinteria, CA, USA) for 20 min. Sections were incubated for 18–20 h at 4 °C with rabbit polyclonal antibodies against TGF-β1 (1:50, OriGene Technologies, Inc., Rockville, MD, USA), β3-adrenoceptor (1:750, Millipore, Temecula, CA), M2 (1:1,000, Millipore, Temecula, CA, USA), M3 (1:1,000, Santa Cruz Biotechnology, Santa Cruz, CA, USA) and IL-6 (1:200, GeneTex, Irvine, CA, USA), mouse monoclonal antibodies against IGF-1 (1:500, Abcam, Cambridge, MA, USA) and BDNF (1:750, OriGene Technologies, Inc. Rockville, MD, USA) and goat polyclonal antibody against TNF-alpha (1:200, R&D Systems, Minneapolis, MN, USA). Then, sections were incubated for 1 h with biotinylated secondary antibody, and avidin-biotin peroxidase was added (1:100, ABC method, PK-6102, Vector Labs, Burlingame, CA, USA). Staining was developed with 3,3’-diaminobenzidine (DAB) plus H_2_O_2_ as chromogen at room temperature. Negative controls were performed using normal, non-immune serum supernatant from the same sources to replace primary antibodies.

### Immunoconfocal microscopy

The neural cell markers in hAFSC culture and the anti-autoimmunity and anti-inflammatory effects of cultured hAFSCs and SCI sections were determined by using immunoconfocal detection^50–52^. For neural cell markers, hAFSCs were plated at 1×10^6^ cells onto glass coverslips in 6-well plates for 2 days and then fixed for 15 min with 4% paraformaldehyde in PBS at room temperature. hAFSCs were permeabilized with 0.5% Triton X-100/PBS for 5 min and then blocked with 10% FBS in 0.1% BSA/PBS for 1 h at room temperature. For SCI sections, following perfusion with 4% paraformaldehyde, spinal cords were dissected, post-fixed overnight in 4% paraformaldehyde, dehydrated for 3 days at 4 °C in 30% sucrose, and embedded in OCT compound. The spinal cord was cryosectioned at a 12 μm thickness and stored at −80 °C. Primary antibodies against hCD90 (1:200, BD Biosciences, San Jose, CA, USA), nestin (1:500, R&D Systems, Minneapolis, MN, USA), β-tubulin III (1:1000, COVANCE, Richmond, CA), GFAP (1:500, DAKO Corp., Carpinteria, CA), BDNF (1:250, Millipore, Temecula, CA, USA), TGF-β1 (1:50, OriGene Technologies, Inc. Rockville, MD, USA), and IL-6 (1:200, GeneTex, Irvine, CA, USA) were used with anti-mouse, anti-rabbit or anti-goat secondary antibodies conjugated with Alexa 488 or 594 (1:500, Invitrogen, Carlsbad, CA, USA). Negative controls were performed without primary antibodies. Stained sections and cell coverslips were mounted onto slides with mounting medium containing DAPI (Santa Cruz Biotechnology, Santa Cruz, CA, USA). A spot charge-coupled device colour digital camera was used to obtain immunohistochemistry images under an Olympus BX-51 microscope (Olympus Corp.) and immunofluorescence under a Leica TCS SP8X confocal laser scanning microscope (Leica Microsystem, Heidelberg, Germany) with the appropriate filters for FITC and DAPI.

Immunoconfocal detection of hAFSCs in SCI sections was performed by staining for hCD90 and DAPI. hAFSC survival was assessed by counting the number of infiltrated hCD90 cells. The number of surviving cells was counted by dividing the spinal cord into 4 areas and manually counting the cell number in each area with 400× micrographs of 5 sections within 1 millimetre in length. These numbers were summed as the total number of surviving hAFSCs per mm^3^.

### Statistical analysis

Data were analysed using one-way analysis of variance followed by Tukey’s test. Values were considered significant at P < 0.05. GraphPad Prism 5 software was used for statistical analysis (GraphPad, San Diego, CA, USA).

## Supplementary information

Supplementary Table and Figure.
