# Supplementary material for: Effect of amniotic fluid stem cell transplantation on the recovery of bladder dysfunction in spinal cord-injured rats
Source: Sci Rep. 2020 Jun 22;10:10030. doi: 10.1038/s41598-020-67163-7 (PMC7308393; doi:10.1038/s41598-020-67163-7)
Supplement: Supplementary file 1 — Supplementary Table and Figure. [file 41598_2020_67163_MOESM1_ESM.pdf]

Effect of amniotic fluid stem cell transplantation on the recovery of bladder dysfunction in spinal cord injured rats

Ching-Chung Liang[1,2], Sheng-Wen Steven Shaw[2,3,4], Yu-Shien Ko[2,5], Yung-Hsin Huang[1] & Tsong-Hai Lee[*,2,6]

[1]Female Urology Section, Department of Obstetrics and Gynecology, Chang Gung Memorial Hospital Linkou Medical Center, Taoyuan, Taiwan

[2]College of Medicine, Chang Gung University, Taoyuan, Taiwan

[3]Division of Obstetrics, Department of Obstetrics and Gynecology, Chang Gung Memorial Hospital Linkou Medical Center, Taoyuan, Taiwan

[4]Prenatal Cell and Gene Therapy Group, Institute for Women's Health, University College London, London, UK

[5]The First Cardiovascular Division, Department of Internal Medicine, Chang Gung Memorial Hospital, Linkou Medical Center, Taoyuan, Taiwan

[6]Stroke Center and Department of Neurology, Chang Gung Memorial Hospital, Linkou Medical Center, Taoyuan, Taiwan

Running title: stem cells improve spinal cord injured bladder

*Correspondence: Tsong-Hai Lee, MD, PhD

Stroke Center and Department of Neurology,

Chang Gung Memorial Hospital, Linkou Medical Center, Taoyuan, Taiwan

No. 5, Fu-Hsing Street, Kweishan, Taoyuan, 333 Taiwan

TEL: 886-3-3281200, ext. 8340; FAX: 886-3-3288849

E-mail: thlee@adm.cgmh.org.tw

Supplementary Table 1. Body and bladder weight in sham treatment, spinal cord injury (SCI) rats with PBS, HEK293 and hAFSC transplantation.

	**Group (N= 6) (Mean ± SD)**	**Body weight (gm; Initial)**	**Body weight (gm; Final)**	**Bladder weight (mg)**
**Day 7**	Sham	264.1 ± 15.3	280.0 ± 10.4	152.5 ± 27.3
	SCI + PBS	276.8 ± 6.6	269.4 ± 15.8	513.8 ± 11.4*
	SCI + HEK293	279.5 ± 7.6	273.7 ± 16.4	523.8 ± 21.4*
	SCI + hAFSCs	284.4 ± 35.0	270.3 ± 39.4	411.7 ± 29.4*#§
	P value	0.3720	0.8602	< 0.0001
**Day 28**	Sham	264.5 ± 10.9	284.6 ± 12.0	158.3 ± 26.0
	SCI + PBS	283.4 ± 20.9	286.2 ± 22.8	438.3 ± 68.5*
	SCI + HEK293	287.1 ± 11.3	290.2 ± 17.9	441.3 ± 39.4*
	SCI + hAFSCs	268.3 ± 12.7	293.8 ± 13.1	415.0 ± 49.6*
	P value	0.0529	0.8130	< 0.0001

* P< 0.0001 vs. control (day 7 and day 28)

# P< 0.0001 vs. SCI + PBS day 7

§ P< 0.0001 vs. SCI + HEK293 day 7

N = 6 rats in each group

Initial body weight: body weight measured before sham treatment, SCI + PBS, SCI + HEK293 or SCI + hAFSC transplantation

Final body weight: body weight measured after sham treatment, SCI + PBS, SCI + HEK293 or SCI + hAFSC transplantation

Bladder weight: bladder weight measured after sham treatment, SCI + PBS, SCI + HEK293 or SCI + hAFSC transplantation

hAFSC = human amniotic fluid-derived stem cell. HEK293 = human embryonic kidney 293 cells. PBS = phosphate buffered saline. SCI = spinal cord injury.

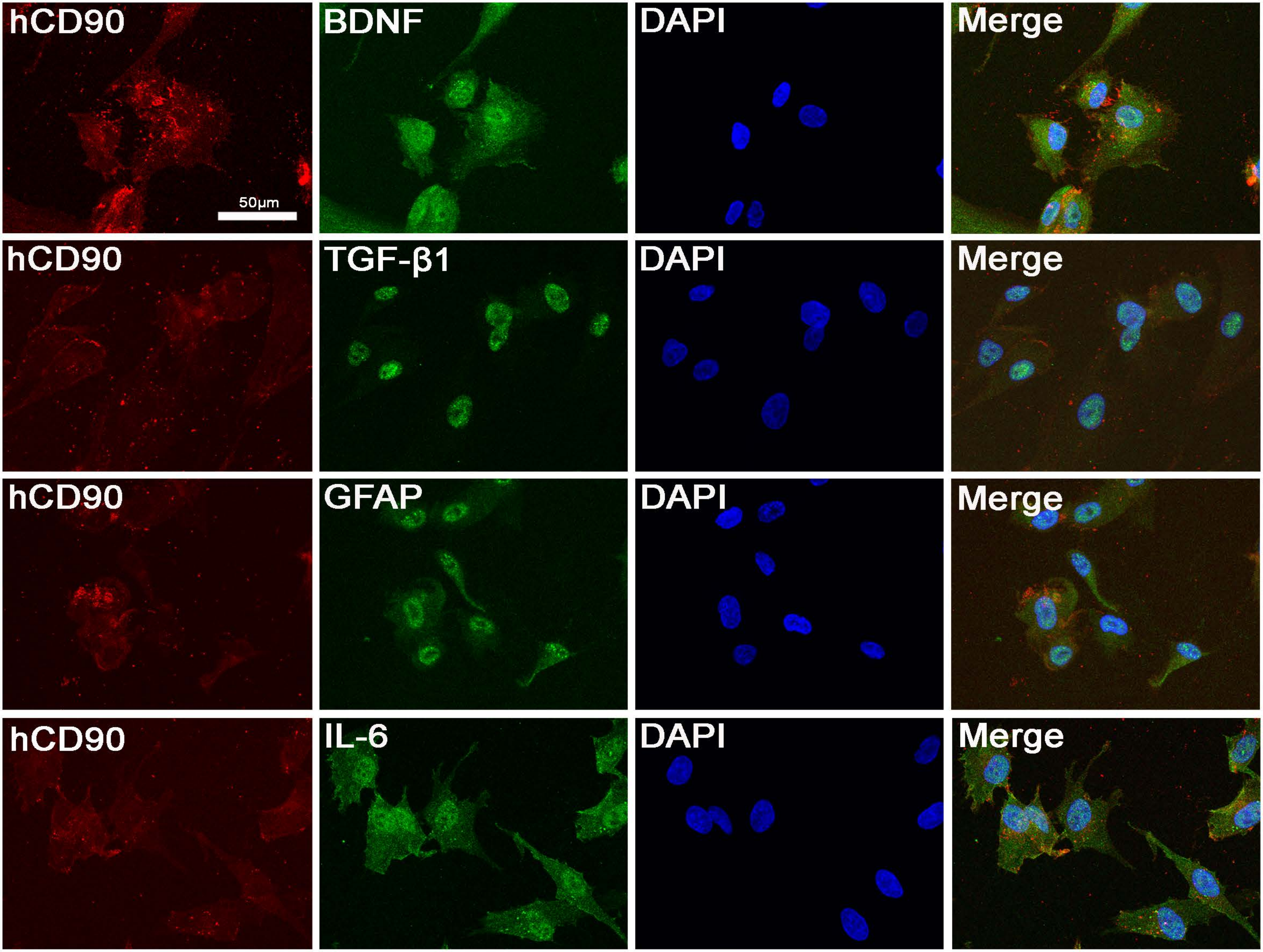
hCD90
BDNF
DAPI
Merge
50μm
hCD90
TGF-β1
DAPI
Merge
hCD90
GFAP
DAPI
Merge
hCD90
IL-6
DAPI
Merge

Supplementary Figure legend

Supplementary Fig. S1.

Immunoconfocal microscopy using triple labeling technique with higher power field than Figure 6 demonstrates colocalization (merge) of DAPI and hCD90 with the neural cell markers BDNF, TGF-β1, GFAP and IL-6 in the spinal cord injured (SCI) section at day 7 after hAFSC transplantation. Bar in hCD90 panel = 50 μm. hCD90 = human CD90. BDNF = brain-derived neurotrophic factor. TGF-β1 = transforming growth factor-beta 1. GFAP = glial fibrillary acidic protein. IL-6 = interleukin-6. DAPI = 4´,6-diamidino-2-phenylindole.

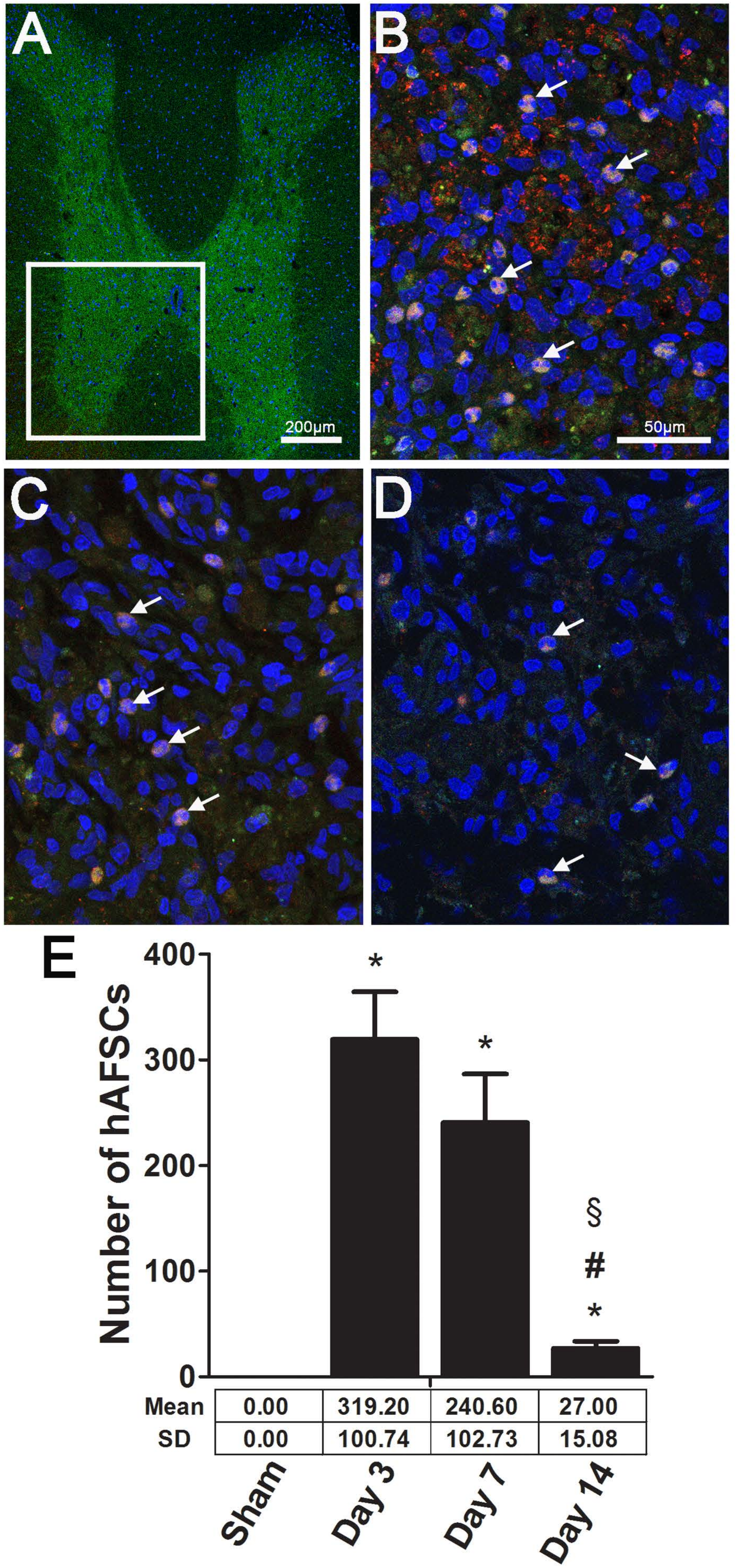
A
B
C
D
200μm
50μm
E
Number of hAFSCs
400
300
200
100
0
*
*
§
#
*
Mean
SD
0.00
0.00
319.20
100.74
240.60
102.73
27.00
15.08
Sham
Day 3
Day 7
Day 14

Supplementary Figure legend

Supplementary Fig. S2.

Immunoconfocal microscopic analysis of the fate of hAFSC transplantation after spinal cord injury (SCI). The cross section of the spinal cord of sham rat is presented in panel A with the asterisk indicating the relative location of panels B-D. The hCD90-positive hAFSCs (arrows, red stain signals) were present at days 3 (B), 7 (C) and 14 (D) after transplantation. The number of hCD90-positive hAFSCs was decreased significantly at day 14 compared to those at days 3 and 7 (E). Bar in panel A = 200 µm. Bar in panel B = 50 µm. N = 6 at each time point. hCD90 = human CD90. DAPI = 4′,6-diamidino-2-phenylindole (blue stain signal).
